# Vault Particles in Cancer Progression, Multidrug Resistance, and Drug Delivery: Current Insights and Future Applications

**DOI:** 10.3390/ijms26041562

**Published:** 2025-02-12

**Authors:** Alexandros Maniatis, Dimitra Rizopoulou, Athanasios-Nasir Shaukat, Katerina Grafanaki, Vassiliki Stamatopoulou, Constantinos Stathopoulos

**Affiliations:** 1 Department of Biochemistry, School of Medicine, University of Patras, 26504 Patras, Greece; a.maniatis@upnet.gr (A.M.); d.rizopoulou@ac.upatras.gr (D.R.); athanasios-nasir.shaukat@nih.gov (A.-N.S.); grafanaki@med.upatras.gr (K.G.); v.stam@upatras.gr (V.S.); 2 Laboratory of Molecular Biology, National Institute of Diabetes and Digestive and Kidney Diseases, Bethesda, MD 20892, USA; 3 Department of Dermatology-Venereology, School of Medicine, University of Patras, 26504 Patras, Greece

**Keywords:** vault particle (VP), major vault protein (MVP), vault RNA (vtRNAs), small vault RNA (svtRNA), cancer progression, multidrug resistance (MDR), drug delivery nanoparticle

## Abstract

Vault particles (VPs) are highly conserved large ribonucleoprotein complexes found exclusively in eukaryotes. They play critical roles in various cellular processes, but their involvement in cancer progression and multidrug resistance (MDR) is the most extensively studied. VPs are composed of the major vault protein (MVP), vault RNAs (vtRNAs), vault poly (ADP-ribose) polymerase, and telomerase-associated protein-1. These components are involved in the regulation of signaling pathways that affect tumor survival, proliferation, and metastasis. MVP has been associated with aggressive tumor phenotypes, while vtRNAs modulate cell proliferation, apoptosis, and autophagy. VPs also contribute to MDR by sequestering chemotherapeutic agents, altering their accumulation in the nucleus, and regulating lysosomal dynamics. Furthermore, small vault RNA-derived fragments participate in gene silencing and intercellular communication, reinforcing the role of precursors of vtRNAs in cancer development. Beyond their biological roles, VPs present a promising platform for drug delivery, due to their unique ability to encapsulate a wide range of biomolecules and therapeutic agents, followed by controlled release. This review compiles data from PubMed and Scopus, with a literature search conducted up until December 2024, highlighting current knowledge regarding VPs and their crucial involvement in cancer-related mechanisms and their applications in overcoming cancer drug resistance.

## 1. Introduction

Vault particles (VPs) are large ribonucleoprotein complexes, with a molecular mass of 13 MDa (three times larger than eukaryotic ribosomes), approximate dimensions of 72.5 nm × 41 nm × 41 nm, and an internal volume of around 5 × 10^4^ nm^3^, making them the largest cellular ribonucleoprotein particle known [1]. They were first identified in 1986 by Kedersha and Rome, and their name (“vaults”) was a token due to their symmetrical, barrel-shaped, featuring multiple arches that resemble cathedral ceilings [2]. Scanning transmission electron microscopy, cryo-electron microscopy, and nuclear magnetic resonance have shown that each VP consists of a hollow, barrel-like structure formed by two identical, cup-like halves joined at their open ends, indicating that the particle can open and close in response to cellular signals [3,4]. VPs primarily consist of the major vault protein (MVP), along with a special class of small non-coding RNAs transcribed by RNA polymerase III (Pol III), known as vault RNAs (vtRNAs), and two additional proteins, vault poly (ADP-ribose) polymerase (vPARP) and telomerase-associated protein-1 (TEP1) [5,6,7]. VPs, found exclusively in eukaryotes, are highly conserved, exhibiting similar sizes, structures, and compositions. They are found in 10^4^ to 10^7^ particles per cell, depending on the cell type and its metabolic state [8,9]. However, they are absent in fungi, plants, and model organisms, like *Saccharomyces cerevisiae*, *Drosophila melanogaster*, and *Caenorhabditis elegans* [10,11].

VPs have been implicated in various cellular functions and their key components, MVP and vtRNAs, emerged as critical modulators of oncogenic signaling, contributing to tumor development [12,13]. The MVP, which constitutes most of the vault complex’s mass, plays a pivotal role in cancer biology. Elevated MVP expression has been correlated with aggressive tumor phenotypes and poor outcome in many malignancies [14,15]. Additionally, MVP is upregulated in various tumors, including colorectal and ovarian carcinomas, where its expression is associated with poor prognosis [16,17]. It has also been shown to modulate key intracellular signaling pathways, such as phosphoinositide 3-kinase (PI3K)/protein kinase B (AKT)/mammalian target of rapamycin (mTOR) and mitogen-activated protein kinase (MAPK), in cell lines including glioblastoma U373, H7, and HAP1. These pathways play critical roles in regulating cell survival, proliferation, and apoptosis, further highlighting MVP’s pivotal role in cancer development and progression [18,19,20].

On the other hand, vtRNAs, once thought to function exclusively within the VP, are now recognized as important modulators of cellular function beyond their structural association with VPs. The four human vtRNA paralogues *vtRNA1-1*, *vtRNA1-2*, *vtRNA1-3*, and *vtRNA2-1* exhibit differential expression patterns across various tissues, tumor types, and stages of cancer progression. Like most Pol III transcripts, vtRNAs play a crucial regulatory role in signaling pathways that govern cell proliferation and apoptosis, making them key contributors to cancer recurrence [10,21,22,23,24]. An even more significant role may be attributed to the poorly studied small vault RNA-derived fragments (svtRNAs), which appear to mediate gene silencing via RNA interference mechanisms like microRNAs (miRNAs). Additionally, their presence in exosomes suggests a critical function in facilitating intercellular communication, contributing to the regulation of key cellular processes in the tumor microenvironment [25,26,27].

Multidrug resistance (MDR) is a major clinical challenge in cancer treatment, referring to the ability of cancer cells to simultaneously resist the effects of multiple, structurally and functionally distinct chemotherapy drugs. It can arise through various mechanisms, including the overexpression of drug efflux pumps, alterations in drug target sites, changes in drug metabolism, and evasion of apoptosis [28]. VPs and their key components MVP and vtRNAs, have been implicated in several aspects of MDR, contributing to the poor efficacy of conventional therapies. It has been reported that VPs can act as cellular “drug depots”, sequestering drugs away from their intracellular targets or preventing their accumulation intracellularly [29]. MVP’s involvement in key signaling pathways, coupled with its role in regulating lysosomal function in resistant cell lines, positions it as a critical regulator of therapeutic resistance [30,31,32,33]. Simultaneously, vtRNAs through their desensitization to chemotherapeutics like mitoxantrone and their involvement in key signaling pathways and autophagy, position them as pivotal players in MDR mechanisms [34,35,36,37].

Herein we provide a comprehensive overview of the current understanding of VPs, starting with their evolutionary origins and the mechanisms governing cargo encapsulation and release, and further delving into the role of VP’s components in critical biological processes such as cancer progression, metastasis, and MDR. Finally, the potential of VPs in drug delivery is highlighted, focusing on their unique structure and ability to encapsulate various therapeutic agents.

## 2. Methods

This review was conducted using a systematic approach by searching the literature on PubMed “https://pubmed.ncbi.nlm.nih.gov/ (accessed on 31 December 2024)” and Scopus “https://www.scopus.com (accessed on 31 December 2024)” databases. The search terms included “vault particle”, “vault nanoparticle”, “vault complex”, “vaults”, “major vault protein (MVP)”, “lung resistance protein (LRP)”, “vault RNAs (vtRNAs)”, “small vault RNA (svtRNA)”, “vault poly (ADP-ribose) polymerase (vPARP)”, “telomerase-associated protein-1 (TEP1)”, “multidrug resistance (MDR)”, “cancer signaling”, “chemotherapy”, and “drug delivery systems”. The search covered the literature published up until December 2024. To improve clarity and readability, a table (Table 1) has been included that provides the full-term names alongside their corresponding abbreviations used throughout this review.

## 3. Structural Insights into VPs

**Figure 1:**
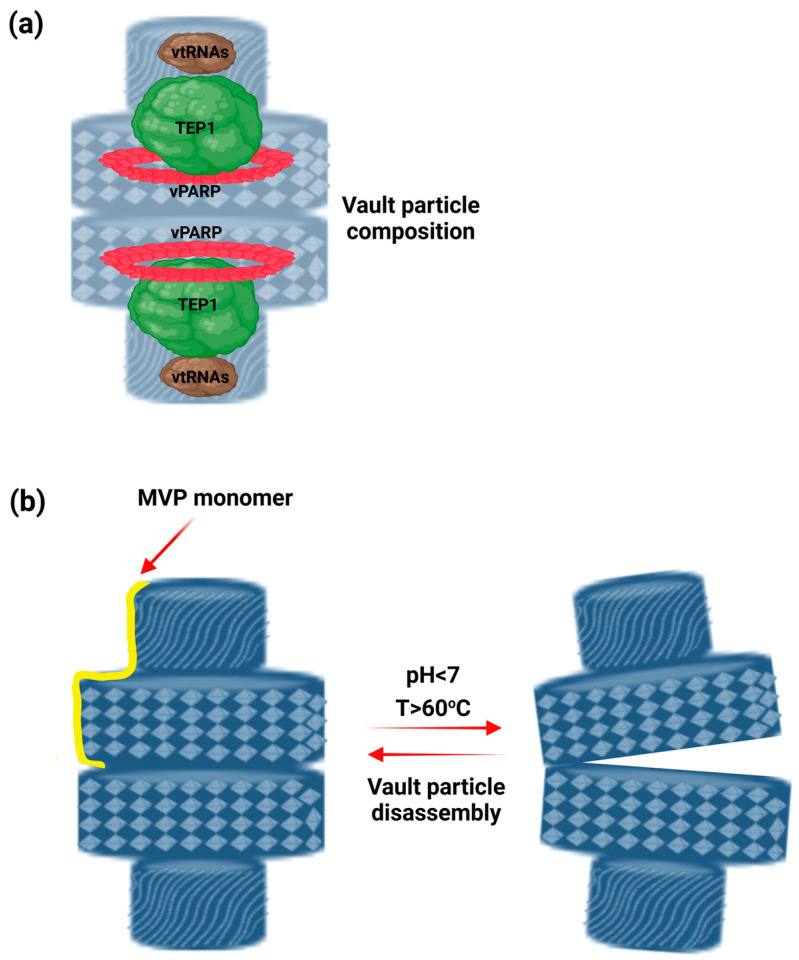
**Architectural characteristics of vault particles (VPs) and their opening mechanism.** (**a**) A cross-sectional view featuring a schematic illustration and the positioning of the VP’s components. The outer shell of the mammalian VP is composed of 78 copies of major vault protein (MVP), which assembles into a large internal cavity that houses vault RNAs (vtRNAs), vault poly (ADP-ribose) polymerase (vPARP), and telomerase-associated protein-1 (TEP1). vtRNAs are occupied at the edge of the particle. TEP1 interacts directly with vtRNAs, and it is essential for their incorporation inside the particle. Meanwhile, vPARP binds internally to the MVP, stabilizing the whole particle, (**b**) VP can encapsulate and release its cargo under specific conditions. In vitro experiments have shown that at pH < 7 and at a temperature above 60 °C, the VP rapidly opens into two halves and releases its delivery.

In mammals, VPs are constructed from a main protein, MVP, which forms a spacious internal cavity wherein vtRNAs, along with two additional proteins, vPARP and TEP1, are included (Figure 1a) [5,6,7].

MVP, a 100 kDa protein, constitutes most of the particle’s mass and can self-assemble into two symmetrical, cup-shaped, hollow halves that form vault-like structures. 

The minimum vault structure, retaining its natural morphology, can be generated by only expressing the MVP in insect cells [38]. Protein Data Bank (PDB) (entry 4V60, https://www.rcsb.org/) provides the X-ray structure of rat MVP that has been solved at a resolution of 3.5 Å. It was suggested that the VP comprises 78 copies of MVP (39 copies per half-vault), with the waist of the particle formed by an end-to-end association of these half-cup-shaped structural domains of monomer MVP. Each monomer consists of 12 domains: 9 structural repeats of β-sheet domains at the N-terminal (R1–R9 domains,) followed by an α/β shoulder domain with 4 α-helices and a 4-stranded β-sheet, a 42-turn-long cap helix, and a C-terminal cap-ring domain. Interactions within the long cap-helix domains play a crucial role in stabilizing the VP. In addition, electron microscopy studies suggested rearrangements in the N-terminus of MVP as the two vault halves open and close, along with a disruption of 39-fold symmetry in the cap region. These findings highlight the vault’s dynamic architecture and mark an essential advancement in understanding the particle’s biology and regulatory mechanisms [29,39,40].

Within the VP, TEP1, a 240 kDa protein, found both in the particle and telomerase complexes directly interacts with vtRNAs and it is essential for their incorporation [41]. Although telomerase activity depends on its catalytic subunit, hTERT, TEP1 itself lacks catalytic function. While the exact function of TEP1 remains uncertain, it is believed to play a role in telomere maintenance and RNA processing due to its presence in both telomerase and vault complexes [42]. In addition, vPARP, a 193 kDa protein, plays a role in stabilizing vtRNAs inside the particle [43]. While vPARP is partially associated with VPs, it also localizes to various cellular compartments, including the nucleus, cytoplasm, and the mitotic spindle during cell division. This distribution suggests a dynamic exchange between free and vault-bound vPARP [4,44,45]. The number of vPARP molecules per vault varies, but the most accepted is approximately nine [46]. Additionally, in vitro studies have shown that vPARP can ADP-ribosylate itself as well as MVP [6].

**Figure 2:**
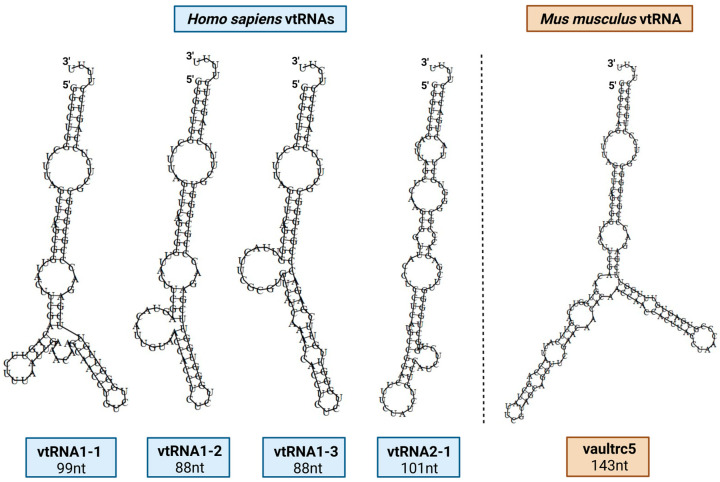
**Predicted secondary structures of the four human vault RNAs (vtRNAs), on the left, and the sole mouse vtRNA, on the right, using RNAFold**.

**Figure 3:**
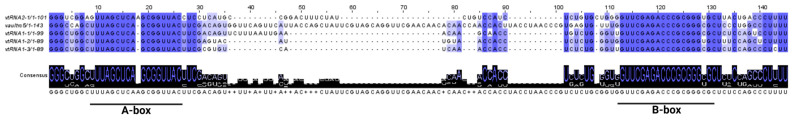
**Sequence alignment of the four human vault RNAs (vtRNAs) and the sole mouse vtRNA.** The alignment was performed using Clustal Omega and Jalview 2.11.1.7 was used for illustration purposes. For the alignment, the highly conserved nucleotides (100%) are indicated by dark blue color, the moderately conserved nucleotides (80%) are indicated with blue color, and the less conserved nucleotides (60%) are indicated with light blue color. It also illustrated the Pol III type 2 promoter, with two internal promoter sequences, the A box, and B box, which facilitate binding with the transcription factors TFIIIC and TFIIIB, like the mechanism in tRNAs. Alignment numbering does not correspond to the numbering of the respective vtRNAs.

vtRNAs are small non-coding RNAs with an average length of 100 nts and constitute less than 5% of the VP’s mass (Figure 2). Most of them (95%) are not associated with VPs and their degradation does not affect their structure. Instead, they distribute within the cytoplasm, where they participate in various cellular processes, including apoptosis, proliferation, immune response regulation, autophagy, and synapse formation [4,7,47,48,49,50]. In humans, the four vtRNAs (Figure 2) are encoded on chromosome 5q31, with three located in the *VTRNA1* locus (vtRNA1-1, vtRNA1-2, and vtRNA1-3) and the fourth (vtRNA2-1) encoded in *VTRNA2* locus [51]. vtRNAs are transcribed under the control of a Pol III type 2 promoter that features two internal promoter sequences (A box and B box) that allow for binding with the transcription factors TFIIIC and TFIIIB, similar to tRNAs (Figure 3) [10]. However, the promoters of the *VTRNA1* and *VTRNA2* loci differ, leading to differences in the expression efficiency of different vtRNA genes [21,22]. vtRNA1-1 is the most abundant vtRNA found in immunoprecipitated VPs from various cell lines, representing 80% of the RNA mass in the complex. The percentage of each of the four vtRNA species found on the vault complex is not proportional to their expression levels, highlighting the differential binding affinity of each vtRNA to the VP [5]. Interestingly, it is proposed that free cytoplasmic vtRNAs undergo processing into svtRNAs. svtRNAs are generated from the full-length vtRNAs by the ribonuclease DICER, following a processing pathway similar to that of miRNA production. The resulting svtRNAs bind to argonaute proteins, the core components of the RNA-induced silencing complex, and function similarly to miRNAs on mRNA silencing [23,25,26,52].

## 4. The Evolutionary Landscape of VPs

VPs are highly conserved across nearly all eukaryotic organisms, exhibiting similar sizes, structures, and compositions, an observation that highlights the importance of their architecture to their function [3]. They have been identified in a wide range of species, including various mammals (human, rat, rabbit, cow, and chicken) and more distantly related organisms like mollusks, echinoderms (*sea urchin Strongylocentrotus purpuratus*), amphibians (*Rana catesbeiana*, *Xenopus laevis*), birds (*Gallus gallus*), and fish (e.g., electric ray *Torpedo marmorata*). To date, no MVP-encoding genes have been identified in fungi, arthropods, or nematodes [42], including certain model organisms, such as *Caenorhabditis elegans*, and *Drosophila melanogaster* and in *Saccharomyces cerevisiae* [4]. Interestingly, in slime mold *Dictyostelium discoideum*, the exception of half-vault structures has been reported, which might stem from the organism’s distinctive amoeboid physiology rather than fundamental species-specific differences [11].

While vtRNA’s sequences are highly conserved (Figure 3), the number of paralogs varies across the animal kingdom. For instance, in addition to the four *VTRNA* paralogs, the human genome includes one confirmed pseudogene, *VTRNA3-1P*, on the X chromosome and another unconfirmed pseudogene, *VTRNA2-2P*, on chromosome 2. The *VTRNA1* locus in humans encodes three paralogs, with orthologs also found in the closely related chimpanzee (*Pan troglodytes*) genome. On the other hand, other primates have only two *VTRNA1* paralogs. Sequence alignment analysis and additional evidence suggest that *VTRNA1-2* and *VTRNA1-3* are the result of a recent gene duplication event, and they contain very similar sequences. Notably, most mammals, including the widely used model organism *Mus musculus*, have only a single copy of *VTRNA1* (vaultrc5) (Figure 2). Except for the sloth (*Capromys hoffmanni*) genome, in which two *VTRNA2* paralogs are present, most mammals either lack the *VTRNA2* locus or encode only one vtRNA paralog. The secondary structure of vtRNAs is generally conserved and comprises an extended stem structure with a central domain of variable structure, known as the “panhandle”. While the handle portion is conserved in different animals and maintains a consistent length, the central domain’s sequence and size vary widely [10].

Bioinformatics analyses employing tools like BLAST, I-TASSER, and RosettaDock have been pivotal in unraveling the evolutionary trajectory of VPs and their components, including MVP, vPARP, TEP1, and vtRNAs. MVP existed in the last eukaryotic common ancestor (LECA), while vPARP, TEP1, and vtRNAs likely followed separate evolutionary paths, with their incorporation into VP occurring later in evolution. The ability of VPs to encapsulate diverse substances and their role in detoxification may have contributed to their retention in certain species while being lost in others [53,54]. The question of how vault-associated molecules may have co-evolved remains a topic requiring further investigation.

## 5. Mechanism of Vault Particle Cargo Encapsulation and Release

The structure of the particle indicates an inherent system for encapsulating and delivering cargo. The VP assembly begins with the lateral interactions among multiple self-oligomerizations of MVP monomers to form half-vault structures. Subsequently, the stabilization of two half particles in an anti-parallel arrangement is mediated by the interaction of N-terminal R1 domains of MVP. This process allows the halves to join and form a complete particle [29]. This mechanism allows them to either release molecules stored inside them or take up molecules from their medium [42].

Recently, extensive mutational analyses of MVPs followed by cryo-EM analyses revealed that VPs transition through five dynamic conformational states during opening. In the closed state, the particle forms a symmetrical structure with MVP R1 domains interacting at a 60° angle, stabilizing the inner cavity for cargo transport. The opening process begins with relaxation at the waist, initiated by localized shifts in certain R1 domains, which increases the angle to about 80°. Once the angle exceeds 97°, the distortion propagates further, forming a gap between R1 domains that ultimately leads to the full separation of the two halves. These structural states closed (60°), relaxed (80°), primed (97°), committed, and opened can transition reversibly. This dynamic behavior underscores the vault’s structural adaptability for efficient cargo encapsulation and release [29].

In vitro experiments have shown that under acidic conditions, VPs rapidly break apart into halves, while at neutral pH, staying intact and fully closed [55]. This pH-dependent opening mechanism could serve as a controlled-release system within cells, leveraging the acidic environment of endosomes and lysosomes for targeted delivery [1]. When examining the effects of pH together with temperature, VPs remained stable at higher pH and below 40 °C, but became unstable at pH 4–5 or temperatures above 60 °C [55] (Figure 1b). However, in vivo experiments reveal that they can partially change conformation at neutral pH, indicating that their opening and closing are not solely pH-dependent [8]. Studies using atomic force microscopy and other biophysical methods show that acidic pH weakens lateral interactions between MVP subunits, rather than affecting the vault’s midsection [56]. These findings suggest that VPs exhibit multiple dynamic conformational states influenced by various yet unidentified factors [39,57].

## 6. Intracellular Localization and Trafficking of VPs

**Figure 4:**
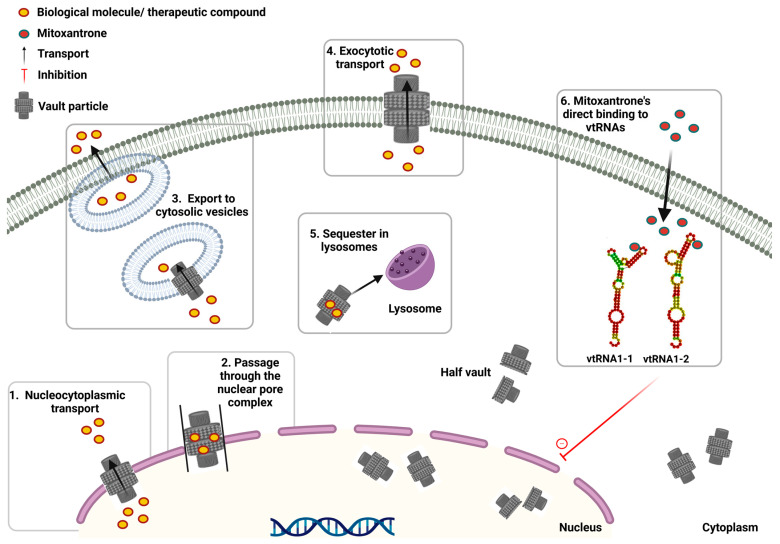
**Mechanisms underlying vault-mediated drug resistance**. Drugs can (1) be transported out of the nucleus, (2) or encapsulated inside vault particles and pass through nuclear pores, (3) accumulate within cytosolic vesicles, (4) undergo exocytosis, (5) become sequestered within lysosomes or (6) can be trapped by directly binding to vtRNAs like mitoxantrone.

Numerous studies have explored the intracellular localization and trafficking mechanisms of VPs. Electron microscopy has revealed that while they are mostly localized in the cytoplasm (>90%), they have also been found at the nuclear pore complex, suggesting their possible involvement in nuclear–cytoplasmic transport (Figure 4) [58]. Studies on cortical neurons reinforce this observation [59].

VPs have also been detected in intranuclear regions, especially near nucleoli. However, the physiological relevance of these interactions remains unclear [60,61]. Their trafficking also involves interactions with cytoskeletal components. In the immortalized PC12 cell line derived from rat pheochromocytoma and in non-small-cell lung cancer (NSCLC) cells, a small portion of the particles was found to associate with intact microtubules, likely supporting the transport of cargo proteins toward the nucleus; they can also move toward the plasma membrane [62,63].

Furthermore, VPs have been linked to lysosomal integrity and vesicular trafficking, suggesting roles in drug disposal and extracellular vesicle secretion [30,64]. They have been observed in diverse cellular locations, like neurotic tips, together with actin filaments, and in lipid rafts of macrophages and of human lung epithelial cells infected with *Pseudomonas aeruginosa* [65,66]. Collectively, these findings shed light on the largely unexplored realm of VP intracellular and extracellular trafficking, which could provide valuable insights into the wide variety of biological functions they involved.

## 7. MVP, vtRNAs, and Their Fragments as Regulators of Cancer Progression

Since their discovery, VPs have been the focus of significant research, revealing their fundamental roles in eukaryotic cell biology. They are implicated in various cellular processes, including cancer development, MDR mechanisms, cell signaling pathways, and immune response mechanisms [43,60].

In 54 colorectal cancer patients, MVP expression was found to be upregulated in metastatic lymph nodes compared to primary tumors. These findings suggest that MVP may play a critical role in the epithelial-to-mesenchymal transition (EMT) and could enhance the migratory activity of metastatic cells [16]. Furthermore, a study with ovarian carcinoma patients revealed a notable discrepancy between MVP transcription and translation levels. Reverse transcription quantitative polymerase chain reaction (RT-qPCR) analysis showed reduced MVP transcription in tumor samples compared to normal ones, whereas immunohistochemistry demonstrated elevated MVP levels, which correlated with higher tumor grades. Interestingly, a similar pattern was observed for the other two VP proteins, TEP1 and vPARP. This discrepancy may be attributed to post-transcriptional regulation, where mRNAs encoding vault components are translated more efficiently in tumors than in normal tissues. Additionally, VP’s proteins might exhibit greater stability in higher-grade cancers, potentially due to their more effective incorporation into VPs [17].

It has been shown that MVP can also be expressed on the surface of a variety of cell lines, including hepatocellular carcinoma (HCC) cells. Cell surface MVP (csMVP) was found to be upregulated in response to stressful conditions, including serum starvation, DNA damage, and detachment stress. Knock-down of csMVP reduced cell proliferation and induced apoptosis. Furthermore, csMVP-positive circulating tumor cells (CTCs) were detected in HCC patients with metastases, suggesting csMVP-positive CTCs might represent a subpopulation with increased survival and metastatic potential [67]. A study in a colon cancer cell line CT26 has linked MVP to exosomes and tumor-suppressor miR-193a. MVP binds to miR-193a, packaging it into exosomes, which lowers its cytoplasmic levels. This leads to increased caprin-1 production (miR-193a targets caprin-1 mRNA), disrupting the cell cycle and promoting cancer growth and metastasis in colon cancer cells [68]. This expanded body of research underscores the complexity of MVP’s involvement in cancer progression.

Most of the information about the implication of vtRNAs in cancer progression comes from vtRNA1-1 [24]. Knock-out (KO) of vtRNA1-1 in cervical adenocarcinoma HeLa and HCC Huh-7 cells exbibits decreased proliferation rates. The phenotype was reversed upon lentiviral overexpression of vtRNA1-1 in both cell lines, indicating that it plays a key role in regulating proliferation [34,69]. The role of vtRNA1-1 in cell proliferation was further validated in Burkitt lymphoma cell lines BL2 and BL41. Overexpression of vtRNA1-1 in these cells resulted in a striking acceleration of growth rates, highlighting its significant contribution to tumor aggressiveness [22,70]. Finally, in MCF-7 cells, vtRNA1-1 was found to interact with the protein-associated splicing factor (PSF), a transcriptional regulator known to repress the proto-oncogene G antigen 6 (GAGE6). By binding to PSF, vtRNA1-1 reduces its affinity for the GAGE6 promoter, thereby alleviating transcriptional repression. This leads to increased GAGE6 expression, ultimately promoting cell proliferation [23]. Studies have also implicated vtRNA1-1 in the regulation of apoptosis in cancer cell lines HeLa, BL41, BL2, A549, and Hs578T through the management of the PI3K/AKT and ERK1/2 MAPK signaling pathways. Notably, it has been indicated that there are some key nucleotides in the central domain of vtRNA1-1 responsible for apoptosis regulation [69,70].

Evidence regarding the role of vtRNA1-2 remains limited. However, it appears to be crucial for cell viability. Notably, studies have reported that the KO of vtRNA1-2 in cell lines is lethal, underscoring its essential function in survival. Data from the Cancer Genome Atlas (TCGA) suggests that vtRNA1-2 may function as a potential tumor suppressor. Its promoter is methylated in many tumors, leading to reduced expression; however, an exception is observed in liver-derived metastases, where vtRNA1-2 expression is significantly elevated [71]. Notably, both vtRNA1-1 and vtRNA1-2 are highly expressed in metastatic liver cancer cells, whereas their expression is undetectable in the surrounding non-malignant tissue. This distinction underscores their potential involvement in metastatic progression events [22].

Although vtRNA1-3 shares evident similarities in primary and secondary structures with vtRNA1-1 and vtRNA1-2, it does not appear to regulate apoptosis or cell proliferation either in Burkitt lymphoma or in HeLa cells [69,70]. However, a common characteristic among these vtRNAs is their elevated expression in drug-resistant cell lines SW1573/2R120 (NCSLC), GLC4/ADR (SLC), MCF-7/MR (breast cancer), 8226/MR4, 8226/MR20 (myeloma cells) [72]. Additionally, in patients with myelodysplastic syndrome and reduced survival rates, hypermethylation of the vtRNA1-3 promoter has been observed [73]. vtRNA1-3 remains poorly studied, and its potential roles in critical biological processes, such as cellular metabolism and cancer progression, are still largely unexplored.

vtRNA2-1, initially identified as pre-miR886 or nc886, was the most recent non-coding RNA discovered to be associated with the VP. It plays a key role in regulating apoptosis and cell proliferation by directly binding to the pro-apoptotic factor protein kinase R (PKR) and inhibiting its phosphorylation [74]. This regulatory function was evidenced in cholangiocarcinoma cells and patients, where decreased vtRNA2-1 expression correlated with increased phosphorylation of PKR. vtRNA2-1 was proposed to contribute to carcinogenesis by activating the PKR-eIF2α pathway, which, in turn, triggers the pro-survival NF-κB signaling cascade [75]. Finally, reduced vtRNA2-1 expression in various cancers has been linked to promoter methylation, which is associated with poor prognosis. Consequently, vtRNA2-1 expression levels could serve as a valuable prognostic marker in cancers such as lung, prostate, esophageal, gastric, and acute myeloid leukemia (AML) [22].

The knowledge regarding the regulatory role of svtRNAs in cancer progression is limited solely to those deriving from vtRNA2-1 (termed as miR-886-3p and miR-886-5p), which display reduced expression in cancerous tissues in comparison to healthy ones, indicating potential role as tumor suppressors [12,25,76]. miR-886-3p downregulates key cancer pathways, like proliferation, migration, and invasiveness in prostate, thyroid, and lung cancer [23,71,77]. However, miR-886-3p was found to be upregulated and to inhibit apoptosis in clear cell renal cell carcinoma. The proposed mechanism was that the fragment targets the mRNA of the transcription factor PITX, a tumor suppressor molecule, which leads to decreased PITX translation and reduced cell apoptosis [77].

Another intriguing function of svtRNAs is their involvement in cell-to-cell communication, as they have been abundantly detected in exosomes, key mediators of tumor microenvironment interaction [77]. Numerous reports have demonstrated exosome-based cell-to-cell communication facilitated by miRNAs that regulate tumorigenesis and angiogenesis [22]. Thus, these pathways may be regulated by svtRNAs secreted through exosomes. Very recently, svtRNA1-2, derived from the 3′ end of vtRNA1-2, has been suggested to interact with specific intronic regions of nascent transcripts like tRNA-derived small RNAs. This interaction can modulate the expression of genes related to the cell membrane and adhesion, processes that are crucial for cancer cell migration and metastasis. The silencing activity of the fragment relies on distinct molecular features, including a 5-nucleotide loop protrusion [27].

## 8. Contribution of VPs and Their Components to Multidrug Resistance

MDR is a major obstacle in cancer treatment; thus, numerous proteins associated with MDR have been identified and several mechanisms, like autophagy and epigenetic changes, have been linked to this phenomenon [78]. The most studied proteins are the ATP-binding cassette (ABC) superfamily that facilitates the export of various compounds in an ATP-dependent manner [79]. Among them, the best known ABCB1 transporters [also named MDR1 or P-glycoprotein (Pgp)] are overexpressed in MDR cells, expelling drugs from tumor cells [80]. However, in cases where ABC transporter-mediated resistance fails, VP, along with their key components MVP, vtRNAs, and svtRNAs, have been shown to play a crucial role in conferring drug resistance [81].

### 8.1. Vault Particle

The discovery that MVP is identical to the lung resistance protein suggested a link between MVP and drug resistance mechanisms and spurred research into its role in MDR. While the exact vault component responsible for this resistance has not been fully understood, MVP and vtRNAs play a key role [82].

It has been suggested that VPs are implicated in MDR phenomena by three main mechanisms: (1) transporting DNA-damaging drugs from the nucleus to cytosolic vehicles, (2) sequestering in lysosomes and, (3) transferring from cytoplasm to exterior through exocytotic transport (Figure 4) [29,42]. One of the earliest discoveries was the interaction of VPs with the estrogen receptor of MCF-7 breast cancer cells. It has been suggested that the VP supports the nuclear transport of the estrogen receptor; thus, when cells were exposed to anti-estrogen therapy, it failed [83].

### 8.2. MVP

Earliest studies have reported that MVP was overexpressed in various non-Pgp MDR cancers like ovarian carcinoma, AML, oral squamous cell carcinoma, and osteosarcoma; it was correlated with poor prognosis of patients and increased potential for metastasis [72,84,85,86,87,88]. Moreover, numerous investigations have unveiled the key role of MVP in chemo and radiation resistance mechanisms in many other cancer types [88]. In bladder cancer, it was proposed that MVP contributes to drug resistance mechanisms by regulating lysosomal function. Knock-down of MVP sensitized UMUC-3 cells to doxorubicin by enhancing nuclear retention of the drug and altering lysosomal organization, causing them to disperse throughout the cytoplasm. This suggests that MVP may not be involved only in transporting doxorubicin, but also in determining its destination within the cell [30]. A recent study has intriguingly established a connection between obesity, increased levels of MVP, and doxorubicin resistance. It is proposed that adipocytes, when co-cultured with human or murine breast cancer cells, can induce resistance by upregulating MVP expression. The underlying mechanism is that MVP reduces the nuclear accumulation of the drug by sequestering it into cytoplasmic vesicles, followed by extracellular expulsion through vesicular secretion [64]. However, a promising strategy to overcome doxorubicin resistance was proposed by using a multifunctional nanocarrier composed of polyamidoamine–hyaluronic acid to co-deliver MVP-siRNA and doxorubicin into MCF-7/ADR breast cancer cells. MVP knock-down enhanced doxorubicin accumulation in the nucleus and ultimately reversed resistance. The study emphasizes the need for effective, sustained delivery of therapeutic agents to overcome cancer resistance [89].

In HCC cells, overexpression of MVP reduced sensitivity to epidermal growth factor receptor (EGFR) inhibitor gefitinib, enhanced AKT phosphorylation, and lowered the expression of genes associated with inflammatory pathways. Conversely, silencing MVP reversed the phenotype. This revealed for the first time that MVP plays a critical role in mediating resistance to EGFR inhibition [90]. Another proposed mechanism for gefitinib resistance links MVP with Y-box binding protein 1 (YB-1) in lung adenocarcinoma HCC827 and PC-9 cells. YB-1 was higher in gefitinib-resistant cells, making them more resistant by activating the AKT pathway and promoting EMT through increased MVP expression [31].

In temozolomide-resistant glioblastoma U251 and LN229 cells, MVP was found upregulated, and when it was silenced, the sensitivity to temozolomide was increased and the sphere formation ability and invasive capacity of the cells were reduced [32]. Similarly, MVP deficiency in the osteosarcoma U2OS-KO cell line results in slower migration and larger spheroid formation, indicating further MVP’s potential involvement in cell adhesion and metastasis [16]. Lastly, in the A549 lung cancer cell line, overexpression of IL-25 increased MVP levels and enhanced resistance to cisplatin. A possible mechanism could involve activation of the NF-κB pathway by IL-25 overexpression, which in turn triggers an inflammatory response that inhibits apoptosis through MVP overexpression [33].

### 8.3. vPARP and TEP1

While MVP has been linked to several MDR phenomena, the role of the minor vault proteins remains poorly understood, partly due to their limited association with the VP. Two studies have revealed the coordinated expression among the three vault proteins, with MVP influencing vPARP and TEP1 levels. In drug-resistant SCLC cells, MVP overexpression increased vPARP and TEP1 levels and in resistant ovarian carcinoma A2780TR cell line reduction in MVP expression led to decreased vPARP levels [91,92]. These observations support the notion that vault proteins may collaborate, at least partially, in fulfilling their roles, although mechanisms remain elusive.

### 8.4. vtRNAs and svtRNAs

Regarding vtRNAs, information on their involvement in drug resistance mechanisms remains scarce. vtRNA1-1 is typically the most abundant vtRNA transcript in cancer cell lines, with its expression often elevated in drug-resistant cells. vtRNA1-1 and vtRNA1-2 have been suggested to mediate resistance to mitoxantrone by directly binding to the drug at well-characterized regions, as demonstrated through in-line probing. This binding reduces the efficacy of the chemotherapeutic compound. Interestingly, no interaction was observed with vtRNA1-3 or vtRNA2-1 (Figure 4) [35,36]. Reduced vtRNA1-1 expression in HCC cell lines has been associated with improved response to sorafenib treatment. Conversely, in the HCC Huh-7 cell line, vtRNA1-1 was suggested to promote autophagy through the MAPK/TFEB signaling pathway, which adjusts the expression of the coordinated lysosomal expression and regulation (CLEAR) network genes. This processes the formation of metabolically active lysosomes (maintaining acidic pH). This mechanism contributes to the development of chemoresistance [34]. vtRNA1-3 expression is elevated in MDR cells, and its association with the VP is enhanced. These findings suggest a potential role for vtRNA1-3 in MDR, although the exact molecular mechanisms underlying this effect remain to be fully elucidated [5,72]. The vtRNA1-1-derived fragment has been implicated in MDR in breast cancer by targeting and down-regulating cytochrome CYP3A4 mRNA, an important detoxifying drug enzyme that metabolizes many chemotherapeutic compounds. This discovery establishes a functional role for svtRNAs, unveiling new insights into their connection with drug resistance mechanisms for the first time, while also broadening the spectrum of small regulatory RNAs [52,93].

**Figure 5:**
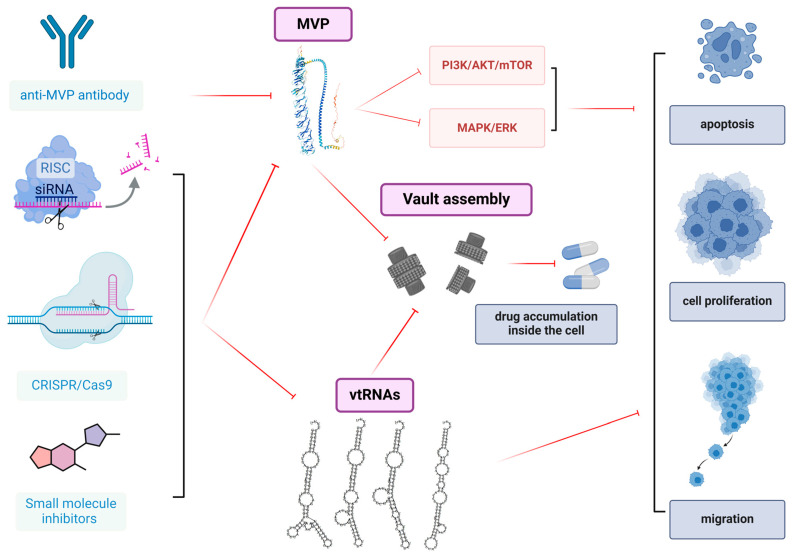
**Potential strategies targeting major vault protein (MVP) and vault RNAs (vtRNAs)**. The *MVP* gene can be knocked out using the CRISPR/Cas9 genome editing system or can be knocked down through RNA interference (siRNA). Additionally, MVP may be targeted by small molecule inhibitors or specific anti-MVP antibodies. These approaches can lead to the downregulation of key cancer signaling pathways, such as PI3K/AKT/mTOR and MAPK/ERK, thereby inhibiting cancer cell proliferation, survival, and migration. Targeting MVP can induce the disassembly of vault particles, allowing therapeutic agents to penetrate and act more effectively within the cell. Similarly, knocking out or knocking down vault RNAs can impair cancer cell proliferation and enhance the accumulation of chemotherapeutic drugs in the cytoplasm, potentially improving treatment efficacy.

Developing strategies to target the VP’s components presents a promising approach that could greatly enhance therapeutic outcomes. By focusing on these key components, it may be possible to disrupt the functions of the VP, thereby enhancing the effectiveness of treatments, overcoming drug resistance, and potentially improving patient response to therapies (Figure 5).

## 9. MVP’s Involvement in DNA Damage Response and Radiotherapy Resistance

Recent studies have highlighted a connection between MVP, not only in MDR but also in DNA damage response and repair mechanisms [15,86,94,95,96]. MVP levels are upregulated in response to DNA-damaging agents, including ionizing radiation, which induces DNA double-strand breaks (DSBs) [97,98]. DSBs are repaired via two major pathways: error-prone non-homologous end-joining (NHEJ) and error-free homologous recombination (HR), and MVP affects both pathways. It correlates inversely with Ku70/80, key NHEJ proteins, and pro-apoptotic BAX, which suppresses NHEJ and promotes genomic instability. This imbalance is associated with altered p53, increased BCL-2, and heightened proliferation, contributing to tumor progression and treatment resistance [99]. In HR, MVP controls Rad51 levels and their movement to the nucleus, which is essential for repairing DNA breaks. When MVP is lost, Rad51 foci decrease, making cells more sensitive to radiation and weakening DNA repair. MVP also helps transport phosphatase and tensin homolog (PTEN) into the nucleus, where it supports Rad51 expression and maintains chromosome stability [100,101]. While impaired DNA repair and disrupted apoptosis promote tumor progression and treatment resistance, MVP can co-regulate genomic stability, making it a potential target for radiotherapy resistance treatment.

## 10. MVP in Cancer Signaling

A key pathway influenced by MVP is the EGF/PI3K/AKT. The PTEN, which inhibits AKT activation by dephosphorylating PIP3, can directly bind to MVP in a calcium-dependent manner [102]. Notably, an intriguing study suggests that MVP facilitates the nuclear import of PTEN [103]. Given that nuclear PTEN induces G1 cell cycle arrest, while cytoplasmic PTEN promotes apoptosis, MVP’s regulation of PTEN’s nuclear–cytoplasmic distribution may play a pivotal role in its anti-apoptotic and tumor-promoting effects observed in glioblastoma [104,105]. Another interesting study has linked MVP with AKT and Notch1 signaling. In the triple-negative breast cancer cell line MDA-MB-231DDPR, Notch1 pathway upregulation partially increased MVP expression, which, in turn, activated the AKT pathway and promoted EMT. The proposed mechanism was that Notch1 could bind to the MVP promoter and increase its expression [106]. Recently, a regulatory link between endoplasmic reticulum stress, PI3K/AKT signaling, and MVP was proposed. Under stress conditions, such as unfolded protein response or tumor hypoxia, PRKR-like endoplasmic reticulum kinase (PERK) undergoes autophosphorylation and becomes activated. However, when inactive, PERK can bind to MVP, blocking PTEN’s nuclear transport and potentially downregulating the PI3K/AKT pathway [18]. Lastly, the overexpression of MVP in cardiac cells exposed to doxorubicin reduced the levels of pro-apoptotic proteins, such as BAX and cleaved caspase-3, and increased the levels of the anti-apoptotic protein BCL-2. This protective effect against apoptosis was accompanied by enhanced phosphorylation of AKT [19]. In human glioblastoma cell lines U373, H7, and RAEW, elevated MVP levels have been associated with increased invasion via PI3K/AKT/mTOR pathway upregulation [104]. Additionally, MVP deficiency in HAP1 *MVP*-KO cells decreased AKT and p53 phosphorylation while also heightening their sensitivity to the anticancer drug vinorelbine, highlighting MVP’s role in chemo-prevention [16].

MVP is also related to the MAPK signaling pathway. Proteomics of senescent MCF-7 cells revealed an upregulation of BCL-2 associated athanogene 3 (BAG3), and mass spectrometry analysis identified interactions between BAG3 and MVP, which can, in turn, regulate apoptosis by altering the phosphorylation status of ERK1/2 [107]. A study in COS-7 cells revealed that MVP can interact with YPEL4. YPEL4 is known to activate a transcriptional activator in the MAPK signaling pathway, named Elk-1. This direct interaction of MVP with YPEL4 significantly reduced the transcriptional activity of Elk-1 and subsequent MAPK signaling pathway downregulation [108]. MVP can regulate the EGFR/MAPK pathway by interacting with ERK and phosphatase SHP-2. When stimulated by EGF, MVP is phosphorylated and binds to these proteins. While it has little effect on ERK activation, it strongly influences Elk-1 signaling. This suggests that MVP acts as a scaffold for SHP-2 and ERK, supporting cell survival [109].

MVP can also regulate the JAK/STAT pathway through a negative feedback loop. When MVP is silent, STAT1 activity and interferon-γ (IFN-γ) production increase, leading to higher MVP expression. In contrast, excessive MVP suppresses STAT1 and IFN-γ, reducing MVP’s promoter activation. This balance helps prevent excessive pathway activation [110]. Similarly, in Lewis lung carcinoma cells, MVP silencing enhanced STAT3 phosphorylation, promoted its nuclear localization, and activated the JAK2 and RAF/MEK/ERK pathways, resulting in increased cell proliferation and reduced apoptosis [111].

Furthermore, it was revealed that MVP plays a crucial role by activating the mTOR/S6K signaling pathway. The transcription factor GLI1, which is overexpressed in chondrosarcoma (CS), can form a complex with MVP and mTOR. This interaction promotes the nuclear localization of GLI1 and enhances its expression by activating the mTOR/S6K1 signaling cascade. Knock-down of MVP reduced cell growth and induced apoptosis in CS cells, and combined inhibition of MVP and GLI1 significantly suppressed CS progression in vitro and in vivo, suggesting MVP is a potential target therapeutic strategy for advanced CS [112].

## 11. MVP and vtRNA in the Regulation of Immune Response

Many investigations highlight MVP’s significant role in influencing the immune response in cancer. A recent analysis across 38 different cancer types highlighted MVP’s critical role in shaping the tumor immune landscape. MVP overexpression in most tumors is positively correlated with immune cell infiltration and a negative association with immunosuppressive cell infiltration, suggesting its significant role in enhancing tumor immune responsiveness [113]. Similarly, in papillary thyroid cancer, high expression of MVP was linked to increased infiltration of various immune cell types, including CD8+ T cells, CD4+ T cells, Th1, Th22, dendritic cells, macrophages, monocytes, neutrophils, natural killer cells, eosinophils, basophils, B cells, and Th2 [20]. A similar pattern was observed also in patients with pancreatic adenocarcinoma [114]. Finally, a study of 119 prostate cancer patients revealed a positive correlation between MVP and the immune checkpoint protein B7-H3, highlighting MVP’s key role in the immunoregulation of cancer [14]. In addition, vtRNA2-1 plays a key role in suppressing IFN-β signaling and regulating inflammation processes. Upon exposure to pathogens, vtRNA2-1 inhibits interferon regulatory factor 3 activation and, through PKR inhibition, also suppresses NF-κB and AP-1. These findings demonstrate that vtRNA2-1 is tightly involved in controlling the expression of IFN-β and the factors required to activate its promoter [115]. A connection could exist between vtRNA2-1 and IFN, as IFN not only plays a role in anti-pathogenic defense but also has anti-tumor properties and was previously regarded as a potential therapeutic agent for cancer treatment [116].

## 12. MVP and vtRNAs in Osmotic Stress, Hypoxia, and Senescence

In SW620 human colon cancer cells, hyperosmotic stress leads to increased MVP expression and upregulation of the PI3K/AKT pathway. While the knock-down of MVP leads to decreased cell viability and increased apoptosis in osmotic stress, the modulation of the pathway may be one of the mechanisms through which MVP protects cells from osmotic stress-induced apoptosis [117]. A study in human renal adenocarcinoma cell line ACHN explored the role of MVP in hypoxia, focusing on hypoxia-inducible factor-1α (HIF-1α). They proposed that both MVP and the whole VP can form a complex with HIF-1a, and under hypoxic conditions, MVP knock-down increased HIF-1α and decreased HIF-1α ubiquitination. This suggests that MVP may act as a scaffold in the HIF-1α degradation pathway, promoting its ubiquitination and subsequent degradation [118]. It has been proposed that MVP levels are elevated in senescent human dermal fibroblasts (HDFs) and aged mouse organs. On the contrary, MVP is downregulated in young HDFs in response to apoptotic stress over time and MVP knock-down increases their sensitivity to apoptosis. The proposed mechanism is that MVP can modulate apoptosis sensitivity in senescent HDFs by elevating levels of the anti-apoptotic protein BCL-2, likely through negative regulation of the JNK/MAPK pathway [119]. Except for MVP, vtRNA2-1 has also been linked to aging. It has been proposed that UVB radiation in keratinocytes reduces vtRNA2-1 expression by increasing PKR phosphorylation via MAPK. This leads to elevated levels of pro-inflammatory cytokines, cyclooxygenase-2, collagenase type IV, and MMP-9, accelerating inflammation and skin aging. However, vtRNA2-1 overexpression inhibited the production of these mediators, suggesting vtRNA2-1 protective role against photoaging [37]. It was later suggested that the downregulation of vtRNA2-1 following UVB exposure was attributed to the methylation of its gene [120].

## 13. MVP as a Biomarker and Drug Delivery System

MVP’s upregulation serves as a prognostic biomarker [113] for chemo and radiotherapy resistance and is associated with poor outcomes across various solid tumors like melanoma [121], ovarian cancer [84], cervical cancer [96], NSCLC [122], oropharyngeal [15], oral squamous cell carcinoma [86], HCC [123], and prostate cancer [98], and blood cancers such as AML [85] and multiple myeloma [13]. Notably, the predictive accuracy is enhanced by integrating MVP upregulation data with the elevated expression levels of other molecules. Thus, designing prognostic or diagnostic tests that detect MVP, and potentially other vault components, could provide a useful prognostic tool. Moreover, recent advancements have focused on designing nanoparticles that can encapsulate and safeguard drugs during administration, subsequently releasing their contents either outside the cell or after cellular uptake. VPs offer a promising enclosing nano-capsule for delivering various cargos (pharmaceuticals, peptides etc.) and developing efficient vaccines against antigens for human pathogens [124,125]. The production of recombinant VPs in *Pichia pastoris* yeast, morphologically identical to those produced in insect cells, achieved solely by expressing complementary DNA (cDNA) encoding MVP, has unlocked new possibilities for their use as advanced delivery vehicles [126]. VPs are highly effective as therapeutic delivery vehicles due to a range of advantageous features. Their spacious internal cavity allows for ample cargo capacity, while their size prevents rapid clearance by the kidney or liver, enabling them to reach draining lymph nodes when administered intradermally. Moreover, they protect encapsulated proteins from degradation by external proteases, and most importantly, they are non-immunogenic. Numerous studies focus on designing VPs by employing genetic and chemical modification methods, particularly targeting the MVP-encoding gene [42]. Firstly, attaching 33-amino-acid residues from the Z domain of protein A to the C-terminal region of MVP creates a strong affinity for VPs with the Fc-binding region of IgG antibodies. This modified VP can bind to any IgG, making it a versatile tool for targeting specific surface antigens [127]. Additionally, incorporating a 12-amino-acid sequence rich in cysteines into the N-terminal region of MVP enhances the stability of the VPs by forming constant disulfide bonds [125]. To improve intracellular drug delivery, a 31-residue segment from the NS5A protein of the hepatitis C virus, containing amphipathic helices, is fused to the N-terminus of MVP. This modification creates lipophilic rings within the particle, enabling the selective binding of hydrophobic drugs. It is estimated that each VP can accommodate thousands of hydrophobic molecules. This strategy lays the groundwork for developing advanced nanodevices aimed at targeted drug delivery in the future [128]. Additionally, attaching a 13-residue cell-penetrating peptide from the TAT protein of HIV1 to the C-terminus of MVP greatly improved cellular uptake [129]. Modifying MVP with polymers such as N-isopropylacrylamide enables temperature-sensitive aggregation, which could facilitate targeted drug release in heated areas [130]. Another polymer variant, N-isopropylacrylamide-co-acrylic acid, was designed to trigger vault aggregation at lower pH levels, making it particularly effective for targeting acidic tumor environments [131]. Other promising therapeutic strategies leverage MVP’s ability to bind proteins through its INT domain. For example, MVPs engineered with immune-stimulating proteins like the major outer membrane protein or PmpG protein from *Chlamydia muridarum* have shown potential as intranasal vaccines in mouse models [132,133]. Finally, VPs fused with the chemokine CCL21 or tumor antigens like NY-ESO-1 have been shown to trigger immune responses in cancer models, resulting in the suppression of tumor growth [134].

Modified VPs can operate through an open-closed mechanism, enabling dynamic and targeted exchange of molecules [42]. However, clinical challenges must still be addressed, including immune response, stability, and efficiency in human trials. VPs’ assembly is suggested to occur spontaneously via the self-oligomerization of MVP monomers, although it may require more yet unidentified proteins. They also exhibit dynamic conformational states influenced by unknown factors, adding extra complexity [29]. Efforts to modify VPs, risk structural instability, and adverse effects, especially when transitioning from simple cell models to more complex environments like mouse models or humans, where unexpected immune responses may arise. These challenges highlight the need for in-depth studies to understand the exact assembly mechanisms of VPs and explore the possible interactions with other molecules. One more significant limitation that should not be overlooked is the fact that the transcription, maturation, and folding processes of vtRNAs remain poorly understood. To preserve their delicate tertiary structure and ensure stability, vtRNAs rely on essential base modifications. However, these modifications pose technical challenges and increase the risk of unexpected misfolding and potential cytotoxic effects. Overcoming these limitations is crucial to unlocking the therapeutic potential of VPs for clinical applications.

## 14. Conclusions

Almost 40 years since their discovery, VPs, the largest known ribonucleoprotein complexes in nature, have emerged as critical players in cancer progression and MDR mechanisms. The ability of VP components, especially MVP and vtRNAs, to regulate apoptosis and proliferation through key cancer signaling pathways underscores their significance as therapeutic targets. Targeting MVP or vtRNA-mediated pathways could enhance cancer sensitivity to conventional treatments that fail and could revolutionize the cancer therapy approach. Additionally, the spacious internal cavity of VPs makes them ideal nanocarriers and an innovative platform for drug delivery. Engineered VPs with peptides, ligands, or polymers, have shown promise in delivering a wide range of therapeutics. Understanding the function of VP components is essential to unlocking their full potential in cancer therapy, and further research can provide valuable insights into their yet uncharacterized roles.

## Figures and Tables

**Table 1:** Abbreviation list.

Full Term	Abbreviation
ATP-binding cassette	ABC
acute myeloid leucemia	AML
The Cancer Genome Atlas	TCGA
chondrosarcoma	CS
cell surface MVP	csMVP
circulating tumor cells	CTC
coordinated lysosomal expression and regulation	CLEAR
cryo-electron microscopy	cryo-EM
double-strand break	DSB
epidermal growth factor	EGF
epithelial-to-mesenchymal transition	EMT
proto-oncogene G antigen 6	GAGE6
hepatocellular carcinoma	HCC
homologous recombination	HR
human dermal fibroblast	HDF
hypoxia-inducible factor-1α	HIF-1α
interferon-γ	IFN-γ
knock-out	KO
last eukaryotic common ancestor	LECA
major vault protein	MVP
mammalian target of rapamycin	mTOR
mitogen-activated protein kinase	MAPK
multidrug resistance	MDR
non-small-cell lung cancer	NSCLC
phosphatase and tensin homolog	PTEN
phosphoinositide 3-kinase	PI3K
protein-associated splicing factor	PSF
protein kinase B	AKT
protein kinase R	PKR
RNA polymerase III	Pol III
small cell lung cancer	SCLC
small vault RNA	svtRNA
telomerase-associated protein-1	TEP1
vault poly (ADP-ribose) polymerase	vPARP
vault RNA	vtRNA
vault particle	VP
Y-box binding protein 1	YB-1
